# Complex chronic patients as an emergent group with high risk of intracerebral haemorrhage: an observational cohort study

**DOI:** 10.1186/s12877-021-02004-4

**Published:** 2021-02-05

**Authors:** Blanca Lorman-Carbó, Josep Lluís Clua-Espuny, Eulàlia Muria-Subirats, Juan Ballesta-Ors, Maria Antònia González-Henares, José Fernández-Sáez, Francisco M. Martín-Luján, on behalf Ebrictus Research Group, Ma Lluïsa Queralt-Tomas, Anna Panisello-Tafalla, Jorgina Lucas-Noll

**Affiliations:** 1 Department of Primary Care, Catalonian Health Institute, EAP Tortosa-est, UUDD Terres de l’Ebre; University Rovira Virgili, Tortosa, Spain; 2 Department of Primary Care, Catalonian Health Institute, University Rovira i Virgili, CAP El Temple, Plaça Carrilet s/n. 43500, Tortosa, Catalunya Spain; 3 Department of Primary Care, Catalonian Health Institute, EAP Amposta, Amposta, Spain; 4 Department of Primary Care, Catalonian Health Institute, EAP Tortosa-est, UUDD Terres de l’Ebre, Tortosa, Spain; 5 Department of Primary Care, Catalonian Health Institute, Fundació Institut Universitari per a la recerca a l’Atenció Primària de Salut Jordi Gol i Gurina (IDIAPJGol), EAP Alcanar-Sant Carles de la Ràpita, Spain; 6 Unitat de Suport a la Recerca Terres de l’Ebre, Fundació Institut Universitari per a la recerca a l’Atenció Primària de Salut Jordi Gol i Gurina (IDIAPJGol), Tortosa, Spain; 7 Department of Primary Care, Catalonian Health Institute; Fundació Institut Universitari per a la recerca a l’Atenció Primària de Salut Jordi Gol i Gurina (IDIAPJGol); University Rovira i Virgili, Reus, Spain

**Keywords:** Cardiovascular, Intracerebral haemorrhage, Complex chronic patient, HAS-BLED score

## Abstract

**Background:**

Demographic aging is a generalised event and the proportion of older adults is increasing rapidly worldwide with chronic pathologies, disability, and complexity of health needs. The intracerebral haemorrhage (ICH) has devastating consequences in high risk people. This study aims to quantify the incidence of ICH in complex chronic patients (CCP).

**Methods:**

This is a multicentre, retrospective and community-based cohort study of 3594 CCPs followed up from 01/01/2013 to 31/12/2017 in primary care without a history of previous ICH episode. The cases were identified from clinical records encoded with ICD-10 (10th version of the International Classification of Diseases) in the e-SAP database of the Catalan Health Institute. The main variable was the ICH episode during the study period. Demographic, clinical, functional, cognitive and pharmacological variables were included. Descriptive and logistic regression analyses were carried out to identify the variables associated with suffering an ICH. The independent risk factors were obtained from logistic regression models, ruling out the variables included in the HAS-BLED score, to avoid duplication effects. Results are presented as odds ratio (OR) and 95% confidence interval (CI). The analysis with the resulting model was also stratified by sex.

**Results:**

161 (4.4%) participants suffered an ICH episode. Mean age 87 ± 9 years; 55.9% women. The ICH incidence density was 151/10000 person-years [95%CI 127–174], without differences by sex. Related to subjects without ICH, presented a higher prevalence of arterial hypertension (83.2% vs. 74.9%; *p* = 0.02), hypercholesterolemia (55.3% vs. 47.4%, *p* = 0.05), cardiovascular disease (36.6% vs. 28.9%; *p* = 0.03), and use of antiplatelet drugs (64.0% vs. 52.9%; *p* = 0.006). 93.2% had a HAS-BLED score ≥ 3. The independent risk factors for ICH were identified: HAS-BLED ≥3 [OR 3.54; 95%CI 1.88–6.68], hypercholesterolemia [OR 1.62; 95%CI 1.11–2.35], and cardiovascular disease [OR 1.48 IC95% 1.05–2.09]. The HAS_BLED ≥3 score showed a high sensitivity [0.93 CI95% 0.89–0.97] and negative predictive value [0.98 (CI95% 0.83–1.12)].

**Conclusions:**

In the CCP subgroup the incidence density of ICH was 5–60 times higher than that observed in elder and general population. The use of bleeding risk score as the HAS-BLED scale could improve the preventive approach of those with higher risk of ICH.

**Trial registration:**

This study was retrospectively registered in ClinicalTrials.gov (NCT03247049) on August 11/2017.

**Supplementary Information:**

The online version contains supplementary material available at 10.1186/s12877-021-02004-4.

## Background

Demographic ageing, which is an almost universal generalized event, has important consequences and repercussions in practically all domains. In healthcare, we are faced with an epidemic of chronic pathologies, disability, and complex health needs, particularly in the older adult population [1]. This phenomenon has led to the emergence of concepts such as ‘comorbidity’, ‘multimorbidity’ and, more recently, ‘clinical complexity’ and ‘complex chronic patient’ (CCP) [2]. The latter is an emerging concept that aims to group intertwined conditions such as multimorbidity, frailty, ageing, and/or sarcopenia. The common element would be the state of clinical vulnerability, which integrates different risk criteria associated with demographic and social evolution. Approximately 4 to 5% of the Catalan population is categorised as CCPs [3] and consumes 65% of health resources. These patients present more frequent and complex interactions with healthcare services, which lead to greater susceptibility to failures in care delivery and coordination. Furthermore, these patients are usually prescribed a broad range of drugs, which leads to poor medication adherence and adverse drug events and interactions. The care of patients with complex chronic conditions is a very challenging area due to the greater and new needs of these patients and offers an opportunity to explore clinical risk assessment modalities and prevention policies, including the identification of vulnerable groups, the analysis of population morbidity and the selection of controls for epidemiological studies [see Additional file 1].

The World Health Organization estimates that the population aged over 60 years will almost double by 2050 [4]. This population group is expected to exhibit an increased incidence of intracerebral haemorrhage (ICH), which is already high in the general population [5]. The incidence of ICH has significantly increased in older adults, which usually present multiple comorbidities, although some peculiarities have been detected depending on the type of population studied. These individuals exhibit a poor prognosis due to the lethality and disability of ICH [6, 7]. The European Stroke Organization and the Stroke Alliance for Europe have proposed decreasing the ICH mortality and improving disability outcomes [8] but have not defined specific preventive actions for the identification and control of predisposing factors or interventions according to risk stratification in specific populations such as CCPs.

Studying ICH in this population group is important for obtaining a better understanding of the disease, identifying methods for its prevention, and aiding treatment decisions because the available evidence of this condition is limited despite its clinical relevance and its associated adverse outcomes. Therefore, the main goals of the present study were to quantify the incidence of ICH in primary healthcare centres and to identify the associated risk factors in a cohort of CCPs.

## Material and methods

### Study design

This is a multicentre, retrospective and community-based cohort study conducted with CCPs who were followed up for 5 years (from 01/01/2013 to 31/12/2017) in primary care centres in the Terres de l’Ebre health area, Catalonia, Spain [see Additional file 2]. This geographical area includes a hospital with secondary care services, 11 primary care teams operating collaboratively with the hospital, a nursing home, and social and mental healthcare services, which conform to a model oriented to coping with the enhanced needs and demands generated by frequent acute exacerbations and intensive use of healthcare services.

### Patients

All patients with an active medical history in any of the participating centres were included in the study. The clinical records should include the condition of CCP at the beginning of the study, and the patients should be living in the study area, including long-term nursing/residential care facilities in the study area. CCP was defined by the presence of at least four of the following criteria [3]: (1) age ≥ 65 years; (2) ≥ 4 active comorbidities; (3) geriatric conditions with functional disability (Barthel Index score < 60, long-term institutionalisation in a nursing/residential care home, or caregivers at home) or recurrent falls; (4) psychosocial disorders (cognitive or psychological disorder with functional disability); (5) active treatment with ≥4 drugs during the previous 6 months; (6) living alone or with a caregiver (≥ 75 years); and (7) use of unscheduled hospital care (two admissions or three emergency room visits due to the exacerbation of chronic diseases) during the previous year.

Patients who had suffered a previous ICH episode or those with a diagnosis of progressive and irreversible advanced chronic disease [3] with a low probability of responding to specific treatments and with a limited life prognosis were excluded. Patients for whom clinical records could not be accessed and/or itinerant or displaced people were also excluded.

### Variables

The main variable was the diagnosis of ICH episodes (coded as I60–70) during the study period. The follow-up time was established from their registration as CCPs in the clinical records until the end of the study, an occurrence of an ICH event, or death from any cause.

We collected the following demographic, clinical, functional, cognitive, and pharmacological variables: age and sex; arterial hypertension (HT) and mean blood pressure (BP; in mmHg and as an average of the measurements obtained in the previous 6 months); cardiovascular diseases, including diagnoses of ischaemic heart diseases, stroke or transient ischaemic accident and/or peripheral arterial disease; and previous falls, presence of cognitive impairment (active diagnosis and/or Pfeiffer’s test > 2 [9]) and/or active prescription of specific medication (antidepressants, sedatives and/or other drugs with effects on the neurological system). The Barthel Index (> 60) [10] and/or the modified Rankin scale (> 4) [11] were used for the assessment of functionality in the basic activities of daily life. The patients were considered institutionalised if they lived in a long-term nursing/residential care home.

The CHA_2_DS_2_-VASC score was available only for AF patients. The risk of bleeding was calculated using the HAS-BLED score [12], regardless of the diagnosis of atrial fibrillation or the prescription of anticoagulant therapy. This measuring scale exhibits good predictive value for intracranial bleeding in anticoagulated patients with atrial fibrillation, although it has also been used for patients without atrial fibrillation and with acute coronary disease [13]. The scale, which has been well validated worldwide and is easy to apply, contains the following variables: age > 65 years; uncontrolled hypertension defined as a mean SBP ≥ 160 mmHg among those with hypertension; abnormal kidney and/or liver function; previous ischaemic stroke; history of bleeding or predisposition; labile INR, defined as time in the therapeutic range during the previous 6 months < 60% (only for patients receiving vitamin K antagonists); and concomitant use of medications (antiplatelet drugs and/or nonsteroidal anti-inflammatory drug [NSAIDs]) and/or alcohol consumption. A HAS-BLED score ≥ 3 indicates an increased risk of bleeding [14]. The HAS-BLED score is considered a useful tool due to the relatively high prevalence of the variables in the CCP population. In addition, this score note the need for regular clinical review and close follow-up and makes clinicians consider potentially reversible bleeding risk factors.

### Data source

The Chronicity Prevention and Care Programme set up by the Health Plan for Catalonia (2011) implemented a new integrated care model for an increasing number of populations with concurrent health and social needs, particularly CCPs with multimorbidity, complexity or advanced chronic disease as well as social needs or dependence [3]. This implementation led to a new scenario where it was possible to work collaboratively with new tools such as integrated health information systems. The Catalan Health Plan extensively implemented a case finding system that classifies high-risk chronic patients into two different categories based on defined criteria and primary care physician judgement: i) complex chronic patients (CCPs, approximately 5% of the population) and ii) patients with advanced chronic disease and a life expectancy of less than 12 months (approximately 1% of the population). The medical records, including the CCP condition, were incorporated into the computerized medical records of the Catalan Health Institute in January 2013. This database is managed by primary care professionals, who administer and update it in a specific format called the “shared individual intervention plan” (PIIC, Catalan acronym for *Pla d’Intervenció Individualitzat Compartit*) [8]. Currently, 82% of individuals registered as CCPs have an updated report.

The cases were identified from clinical records encoded with ICD-10 (10th version of the International Classification of Diseases) in the e-SAP database of the Catalan Health Institute. The Department of Information and New Technologies performed an automated extraction of the CMBD of hospital discharges and SIRE (Integrated Electronic Prescription System, Catalan acronym for *Sistema Integrat* de *Recepta Electrònica*). All the data were included in an ad hoc repository, which was delivered to the main researcher in a completely anonymous format, supervised and assessed according to the General Data Protection Regulation of Spain/Europe of 1st February 2017. For this type of study, formal consent is not required, and the requirement for informed patient consent was waived prior to the inclusion of their medical data in this study. The datasets generated during and/or analysed during the current study are available in the public repository (10.1007/s12325-019-01206-y).

### Statistical analysis

A descriptive analysis was performed using frequencies and percentages for the qualitative variables and means with standard deviations for the continuous variables. This analysis was stratified by group according to whether the patients had suffered from ICH. We assessed differences in proportions and used the nonparametric Mann-Whitney *U*-test to detect significant differences between the two groups. The total incidence density of ICH was calculated by sex and adjusted for age groups. We calculated the incidence rate ratio for ICH risk factors among the exposed portion of the population compared with the unexposed portion. The independent risk factors were obtained from logistic regression models, and the variables included in the HAS-BLED score were ruled out to avoid duplication effects. All variables with a significant *p*-value ≤0.05 for basal differences between the group with ICH and that without ICH were introduced in the multivariate model, which considered those variables associated with a higher risk of ICH based on the available evidence [15] and its clinical significance regardless of its removal by the procedure. The results are presented as odds ratios and 95% confidence intervals (CIs). The analysis with the resulting model was also stratified by sex. All the analyses were performed using SPSS 24.0 statistical software.

## Results

The present study assessed 3594 cases registered as CCP, and 55.2% of these subjects were women. The mean age of the participants was 84 ± 11 years. A total of 271 patients (7.5%) were institutionalised at the beginning of the study. During the follow-up period, 60 patients (1.7%) moved outside the area of study, and 1692 (47.1%) died. The baseline characteristics of this population are illustrated in Table 1 according to whether they experienced an ICH episode during the follow-up period.

**Table 1:** Baseline characteristics of CCP^a^ population according to whether or not they had ICH^b^ during follow-up (01/01/2013–31/12/2017)

	Total	Without ICH^b^	With ICH	p*
	(***N*** = 3594)	(***N*** = 3433)	(***N*** = 161)	
**Age**	84.4 [11.5]	84.3 [11.6]	86.5 [8.7]	0.063
< 80 years (n 840, 23.4%)	70.5 [9.5]	68.1 [11.4]	72.2 [8.8]	0.004
≥ 80 years (n 2754, 76.6%)	89.4 [5.5]	89.3 [5.4]	89.5 [5.5]	0.638
Sex (women)	1985 (55.2)	1895 (55.2)	90 (55.9)	0.925
**Cardiovascular risk factors**				
Arterial hypertension	2707 (75.3)	2573 (74.9)	134 (83.2)	0.017
Diabetes	1510 (42.0)	1452 (42.3)	58 (36.0)	0.115
Hypercholesterolemia	1717 (47.8)	1628 (47.4)	89 (55.3)	0.051
**Comorbidities**				
Cardiovascular disease	1050 (29.2)	991 (28.9)	59 (36.6)	0.034
Ischemic cardiopathy	634 (17.6)	601 (17.5)	33 (20.5)	0.331
Ischemic stroke/Transient ischemic accident	275 (7.7)	259 (7.5)	16 (9.9)	0.264
Peripheral artery disease	306 (8.5)	289 (8.4)	17 (10.6)	0.342
Atrial fibrillation	998 (27.8)	947 (27.6)	51 (31.7)	0.257
Heart failure	947 (26.3)	913 (26.6)	34 (21.1)	0.123
Thromboembolism	294 (8.2)	281 (8.2)	13 (8.1)	0.960
Chronic kidney disease	893 (24.8)	857 (25)	36 (22.4)	0.455
Chronic liver disease	58 (1.6)	57 (1.7)	1 (0.6)	0.306
**Other conditioning factors**				
Institutionalized	271 (7.5)	262 (7.6)	9 (5.6)	0.338
Record of previous falls	508 (14.1)	487 (14.2)	21 (13.0)	0.684
Cognitive impairment or dementia	758 (21.1)	720 (21.0)	38 (23.6)	0.424
Alcohol consumption	506 (14.1)	487 (14.2)	19 (11.8)	0.395
Falls (background)	508 (14.3)	487 (14.1)	21(13.0)	0.395
Bleeding predisposition	285 (7.9)	272 (7.9)	13 (8.1)	0.945
**Clinical data**				
Systolic blood pressure (mmHg)	129.8 [16.2]	129.6 [16.5]	132.8 [13.4]	0.032
Glycosylated haemoglobin A1c (%)	6.6 [2.9]	6.6 [3.0]	6.3 [1.4]	0.247
Glomerular filtration rate (mL/min/1.73m^2^)	56.8 [23.6]	56.4 [23.7]	66.5 [20.9]	0.093
CHA_2_DS_2_-VASC score**	4.6 [1.5]	4.6 [1.5]	4.3 [1.5]	0.669
HAS-BLED score	3.5 [1.1]	3.3 [0.0]	3.5 [0.9]	0.029
Barthel index	70.2 [28.7]	70.1 [28.9]	72.4 [23.7]	0.988
**Medication**				
Oral anticoagulant (VKA^c^ o NOAC^d^)	1050 (29.2)	996 (29.0)	54 (33.5)	0.217
NOAC^d^	106 (2.9)	103 (3.0)	3 (1.9)	0.405
NSAID^e^	2723 (75.8)	2611 (76.1)	112 (69.6)	0.060
Antiplatelet drug	1920 (53.4)	1817 (52.9)	103 (64.0)	0.006
Statin	2006 (55.8)	1921 (56.0)	85 (52.8)	0.430
SSRI^f^	1252 (34.8)	1193 (34.8)	59 (36.6)	0.622
PPI^g^	3144 (87.5)	3008 (87.6)	136 (84.5)	0.238

Data are presented as number of patients (%) or mean [standard deviation] depending on the type of variable, whether qualitative or continuous, respectively. (*) The *p-*value corresponds to the contrast of differences in proportions for qualitative variables and the non-parametric test of the Mann-Whitney U. (**) CHA_2_DS_2_-VASC score only in patients with atrial fibrillation

^a^*CCP* complex chronic patient, ^b^*ICH* intracerebral haemorrhage, ^c^*VKA* vitamin K antagonists, ^d^*NOAC* new oral anticoagulant, ^e^*NSAID* non-steroidal anti-inflammatory drug, ^f^*SSRI* selective serotonin reuptake inhibitors, ^g^*PPI* Proton pump inhibitor

In total, 161 (4.4%) CCPs suffered an ICH episode, and 55.9% were women. The mean age of these individuals was 86.5 (±8.7) years, and women were significantly older (*p* <  0.001). The comparison of these patients with those without ICH indicated relevant differences with respect to the following factors: history of HT (83.2% vs. 74.9%; *p* = 0.017); hypercholesterolemia (55.3% vs. 47.4%; *p* = 0.051); prevalence of cardiovascular disease of any type (36.6% vs. 28.9%; *p* = 0.034); and increasing use of antiplatelet drugs (64.0% vs. 52.9%; *p* = 0.006). The most prescribed drugs were proton pump inhibitors (84.5% vs. 87.6%; *p* = 0.238) and NSAIDs (69.6% vs. 76.1%; *p* = 0.060). An HAS-BLED score ≥ 3 was found in 93.2% of the group with ICH vs. 79.81% of the group without ICH (*p* <  0.001). The average HAS-BLED score of the ICH group was 3.5 ± 0.9, whereas that of the group without ICH was 3.3 ± 0.0 (*p* = 0.029). The overall mortality of the total population was high (47.1%), but a significantly higher mortality was found in the ICH group (58.4% vs. 46.5%; *p* = 0.003).

Differences were found between the sexes (Table 2). The analysis of the characteristics of patients with ICH revealed a higher prevalence of cardiovascular diseases in men (49.3% vs. 26.7%; *p* = 0.003), particularly ischaemic heart disease (31.0% vs. 12.2%; *p* = 0.003), chronic kidney disease (33.8% vs. 13.3%; *p* = 0.002), alcohol consumption (19.7% vs. 5.6%; *p* = 0.006), bleeding predisposition (14.1% vs. 3.3%; *p* = 0.013) and use of antiplatelet drugs (76.1% vs. 54.4%; *p* = 0.005). Men also showed a higher HAS-BLED score (3.7 vs. 3.3; *p* = 0.014). In contrast, women exhibited higher frequencies of hypercholesterolemia (64.4% vs. 43.7%; *p* = 0.008) and falls (17.8% vs. 7%, *p* = 0.045) and an increased use of selective serotonin reuptake inhibitors (46.7% vs. 23.9%; *p* = 0.003).

**Table 2:** Comparison of the characteristics of CCP^a^ population with ICH^b^ according to sex

	Men	Women	p*
	(***N*** = 71)	(***N*** = 90)	
**Age (years)**	83.6 [9.1]	88.9 [7.7]	< 0.001
< 80 years	72.1 [6.9]	72.6 [7.0]	0.861
≥ 80 years	87.8 [5.5]	90.7 [5.3]	0.003
**Cardiovascular risk factors**			
Arterial hypertension	60 (84.5)	74 (82.2)	0.700
Diabetes	30 (42.3)	28 (31.1)	0.144
Hypercholesterolemia	31 (43.7)	58 (64.4)	0.008
**Comorbidities**			
Cardiovascular disease	35 (49.3)	24 (26.7)	0.003
Ischemic cardiopathy	22 (31.0)	11 (12.2)	0.003
Ischemic stroke/Transient ischemic accident	8 (11.3)	8 (8.9)	0.616
Peripheral artery disease	8 (11.3)	9 (10.0)	0.795
Atrial fibrillation	17 (23.9)	34 (37.8)	0.061
Heart failure	13 (18.3)	21 (23.3)	0.438
Thromboembolism	4 (5.6)	9 (10.0)	0.313
Chronic kidney disease	24 (33.8)	12 (13.3)	0.002
Chronic liver disease	1 (1.4)	0 (0.0)	0.259
**Other conditioning factors**			
Institutionalized	2 (2.8)	7 (7.8)	0.174
Record of previous falls	5 (7.0)	16 (17.8)	0.045
Cognitive impairment or dementia	13 (18.3)	25 (27.8)	0.160
Alcohol consumption	14 (19.7)	5 (5.6)	0.006
Bleeding predisposition	10 (14.1)	3 (3.3)	0.013
**Clinical data**			
Systolic blood pressure (mmHg)	134.7 [12.2]	131.3 [14.3]	0.096
Glycosylated haemoglobin A1c (%)	6.6 [1.5]	6.1 [1.3]	0.039
Glomerular filtration rate (mL/min/1.73m^2^)	66.9 [25.3]	65.9 [16.7]	1.000
CHA_2_DS_2_-VASC score	3 [0.00]	5.5 [0.7]	0.102
HAS-BLED score	3.7 [1.0]	3.3 [0.7]	0.014
Barthel index	70.3 [23.7]	75.6 [24.7]	0.145
**Medication**			
Oral anticoagulant (VKA^c^ o NOAC^d^)	22 (31.0)	32 (35.6)	0.542
NOAC^d^	2.0 (2.8)	1.0 (1.1)	0.427
NSAID^e^	49 (69.0)	63 (70.0)	0.893
Antiplatelet drugs	54 (76.1)	49 (54.4)	0.005
Statin	37 (52.1)	48 (53.3)	0.878
SSRI^f^	17 (23.9)	42 (46.7)	0.003
PPI^g^	62 (87.3)	74 (82.2)	0.375

Data are presented as number of patients (%) or mean [standard deviation] depending on the type of variable, whether qualitative or continuous, respectively. (*) The *p-*value corresponds to the contrast of differences in proportions for qualitative variables and the non-parametric test of the Mann-Whitney U. (**) CHA_2_DS_2_-VASC score only in patients with atrial fibrillation

^a^*CCP* complex chronic patient, ^b^*ICH* intracerebral haemorrhage, ^c^*VKA* vitamin K antagonists, ^d^*NOAC* new oral anticoagulant, ^e^*NSAID* non-steroidal anti-inflammatory drug, ^f^*SSRI* selective serotonin reuptake inhibitors, ^g^*PPI* Proton pump inhibitor

The ICH incidence stratified by age group is shown in Table 3. The overall incidence density of ICH was 151/10000 person-years [95% CI 127–174], and a higher density was found in men than in women, although this difference did not reach significance (153 [95% CI 121–184] vs. 148 [95% CI 114–182], respectively). The incidence rate ratios (IRRs) for ICH risk factors are shown in Table 4**.** HT, cardiovascular disease, HAS-BLED score ≥ 3 and treatment with antiplatelet drugs were associated with a higher rate of ICH, but only the HAS-BLED score, hypercholesterolemia and cardiovascular disease were identified as independent risk factors for ICH episodes. The independent ICH risk factors for the CCP population and their stratification by sex are shown in Table 5. The criterion HAS-BLED ≥3 (OR 3.54; 95% CI 1.88–6.68; *p* < 0.001) was the most important risk factor in both women (OR 4.22; 95% CI 1.66–10.71; *p* = 0.002) and men (OR 2.94; 95% CI 1.23–7.05; *p* = 0.015). Additionally, hypercholesterolemia (OR 1.62: 95% CI 1.11–2.35; *p* = 0.012) and cardiovascular disease (OR 1.48; 95% CI 1.05–2.09; *p* = 0.026) were significant in women (OR 2.09; 95% CI 1.24–3.51; *p* = 0.006) and in men (OR 2.15; 95% CI 1.28–3.61; *p* = 0.004), respectively. In contrast, heart failure (OR 0.61: 95% CI 0.41–0.92; *p* = 0.018) and treatment with statins (OR 0.62; 95% CI 0.42–0.91; *p* = 0.015) were associated with lower risk in the entire CPP population but not in the different sexes.

**Table 3:** ICH^a^ incidence by age groups in CCP^b^ population

Age (years)	General Population*	CCP number	HAS-BLED score average	CCP with HAS-BLED score ≥ 3	ICH episodes	ICH Incidence 10,000 person-years
0–14	24,099	3	0.7 [0.6]	1	0	–
15–44	60,289	33	0.9 [0.6]	1 (03.0)	0	–
45–64	50,259	182	1.9 [1.1]	49 (26.9)	5	1 [0–2]
65–74	19,104	317	2.8 [1.4]	250 (78.9)	9	87 [40–166]
75–84	13,231	903	3.3 [1.2]	792 (87.7)	38	116 [82–159]
≥85	7839	2156	3.4 [1.0]	1797 (83.3)	123	166 [138–198]
All	174,821	3594	3.4 [1.1]	2890 (80.4)	161	151 [127–174]

Data are presented as number of patients (%), mean [standard deviation] or value [95% confidence interval], depending on the type of variable, whether qualitative or continuous, respectively. (*) General population in the area of study, Terres de l’Ebre, Catalonia, Spain [see Additional file 2]

^a^*ICH* intracerebral haemorrhage, ^b^*CCP* complex chronic patient

**Table 4:** Incidence Rate Ratio for ICH^a^ risk factors in the CCP^b^ population

Age	Incidence 10,000 person-years	Incidence Rate Ratio (exposed vs unexposed)
≥ 80 years	101 (85–120)	1.5 (0.9–2.2)
< 80 years	69 (46–100)	
**Cardiovascular risk factors**		
Arterial hypertension		
Yes	99 (83–117)	1.6 (1.1–2.5)
No	61 (40–89)	
Diabetes		
Yes	384 (292–497)	0.7 (0.6–1.1)
No	99 (81–120)	
Hypercholesterolemia		
Yes	104 (83–128)	1.4 (0.9–1.8)
No	77 (60–97)	
**Comorbidities**		
Cardiovascular disease^*^		
Yes	112 (86–145)	1.4 (1.0–1.9)
No	80 (65–97)	
Ischaemic cardiopathy		
Yes	98 (67–138)	1.2 (0.8–1.8)
No	86 (72–103)	
Peripheral arterial disease		
Yes	111 (65–178)	1.3 (0.7–2.1)
No	88 (74–103)	
Ischaemic stroke		
Yes	116 (67–189)	1.3 (0.8–2.2)
No	88 (74–103)	
Heart Failure		
Yes	72 (50–100)	0.7 (0.5–1.1)
No	96 (80–114)	
Falls (background)		
Yes	83 (51–126)	0.9 (0.6–1.4)
No	91 (76–107)	
**HAS-BLED score**		
≥ 3	104 (88–122)	3.3 (1.8–6.1)
< 3	31 (16–56)	
**Medication**		
NOAC^c^		
Yes	57 (12–165)	0.4 (0.1–1.4)
No	130 (110–151)	
VKA^d^		
Yes	103 (77–134)	1.2 (0.9–1.7)
No	84 (69–102)	
Antiplatelet drugs		
Yes	107 (88–130)	1.5 (1.1–2.1)
No	69 (53–90)	
Statins		
Yes	85 (68–105)	0.9 (0.6–1.2)
No	96 (75–120)	

Data are presented as value (95% confidence interval). (^*^) Cardiovascular disease includes ischemic cardiopathy and/or peripheral arterial disease and/or ischemic stroke and/or transient ischemic accident

^a^*ICH* intracerebral haemorrhage, ^b^*CCP* complex chronic patient, ^c^*NOAC* direct oral anticoagulant, ^d^*VKA* vitamin K antagonists

**Table 5:** Independent ICH^a^ risk factors for the entire CCP^b^ population and by sex

	Total	Women	Men
HAS-BLED score ≥ 3	3.5 (1.9–6.7)	4.2 (1.7–10.7)	2.9 (1.2–7.1)
Hypercholesterolemia	1.6 (1.1–2.4)	2.1 (1.2–3.5)	1.2 (0.7–2.1)
Cardiovascular disease*	1.5 (1.1–2.1)	1.1 (0.6–1.7)	2.2 (1.3–3.6)
Statins	0.6 (0.4–0.9)	0.6 (0.4–1.0)	0.6 (0.3–1.1)
Heart failure	0.6 (0.4–0.9)	0.7 (0.4–1.1)	0.5 (0.3–1.0)
NOAC^d^	0.5 (0.1–1.6)	0.2 (0.0–1.6)	0.9 (0.2–3.9)
VKA^c^	1.2 (0.9–1.7)	1.6 (1.0–2.6)	0.9 (0.5–1.6)
Falls (background)	0.9 (0.5–1.4)	0.9 (0.5–1.5)	0.7 (0.3–1.9)
Age ≥ 80 years	1.3 (0.9–2.1)	2.3 (1.2–4.6)	0.9 (0.6–1.6)
Diabetes	0.7 (0.5–1.0)	0.6 (0.4–1.0)	0.9 (0.5–1.5)

Data are presented as *odds ratio* (95% confidence interval)

(*) Cardiovascular disease includes ischemic cardiopathy and/or peripheral arterial disease and/or ischemic stroke and/or transient ischemic accident)

^a^*ICH* intracerebral haemorrhage, ^b^*CCP* complex chronic patient, ^c^*VKA* vitamin K antagonists, ^d^*NOAC* direct oral anticoagulant

A HAS-BLED score ≥ 3 showed high sensitivity [0.93; 95% CI 0.97–0.89], a negative predictive value [0.98; 95% CI 0.83–1.12)], a low attributable risk [0.036; 95% CI 0.024–0.048] and a ROC curve with low accuracy [0.567; 95% CI 0.526–0.608]. A HAS-BLED score ≤ 2 showed the best Youden index (94.4).

## Discussion

The results of the present study indicate a higher incidence of ICH in an emerging and vulnerable population subgroup, such as CCPs, than in both general and elderly populations. Data on time trends for ICH in the general population indicate no significant changes in the incidence of ICH over the last two decades [6, 15], but the incidence density of ICH was 5-to-60-fold higher than that observed in the general population, both within the study area and worldwide (Table 6) [6, 16–24]. Comparisons between studies are difficult due to several interacting and overlapping emerging risk factors and aetiologies, new imaging techniques, demographic changes, comorbidities and associated treatments, different target populations, and the lack of a standardized methodology for data recording and exploitation, which makes it impossible to make adjustments to the incidences for different series of patients. Progressive demographic ageing and the inherent characteristics of CCPs could explain these results in ICH incidence [25]. Nevertheless, in the near future, the ICH incidence might be influenced by increases in the percentage of prescriptions of new anticoagulants (NOACs) and improvements in the cardiovascular risk approach.

**Table 6:** Comparative ICH^a^ annual incidence per 10,000 person/years

Author (year)	Studied population	ICH^**a**^ incidence 10,000 person-years	
Current study (2020)	CCP^b^ population	150.6	[127.3–174.7]
	< 80 years	111.6	[111.5–111.6]
	≥80 years	162.4	[162.4–162.5]
González (2016) [18]	> 18 years	2.3	[2.1–2.7]
Carlsson (2016) [19]	≥75 years	24.2	[19.5–28.9]
Jolink (2015) [20]	75–94 years	17.6	[11.0–28.0]
Krishnamurthi (2014) [21]	All ages		
	-High-income countries	4.8	[4.5–5.2]
	-Low-income countries	8.1	[7.3–9.3]
Stein (2012) [22]	≥80 years	12.5	[11.0–14.04]
Van Asch (2010) [6]	> 45 years	2.4	[1.9–3.1]
Lovelock (2007) [23]	≥75 years		
	OCSP^c^	15.5	[9.6–21.3]
	OXVASC^d^	14.4	[9.5–19.2]
Sudlow (1997) [24]	45–84 years		
	Total	3.0–7.9	[2.1–11.4]
	PICH^e^	2.6–6.0	
	SAH^f^	0.4–1.9	

Data are presented as value [95% confidence interval]

^a^*ICH* intracerebral haemorrhage, ^b^*CCP* complex chronic patient, ^c^*OCPS* Oxford Community Stroke Project, ^d^*OXVASC* Oxford Vascular Study, ^e^*PICH* Primary intracerebral haemorrhage, ^f^*SAH* Subarachnoid haemorrhage

Little information about sex-related differences in ICH is currently available, but it appears that the incidence of ICH could be influenced by interactions between sex and other factors. The present study showed some sex-related clinical differences in a large database of ICH patients and thus adds accurate data to a topic for which limited information is available in the current literature, such as a higher prevalence of antiplatelet drug therapy among men. However, unlike the results obtained by other researchers, no significant differences in the incidence of ICH were found after sex stratification [26]. These data contrast with the results from a systematic review of 17 epidemiological studies, which reported a higher overall incidence of ICH in men [27]. Most likely, the presence of sex differences in risk factors encourages us to think about different approaches and the need for specific methods to assess the risk for ICH in CCPs.

Among the multiple ICH risk factors [5, 28], those eligible for intervention stand out: HAS-BLED score ≥ 3, hypercholesterolemia and cardiovascular disease independently increased the risk of ICH, whereas the use of statins and the diagnosis of heart failure were identified as protective factors. These results reflect an insufficient control of modifiable risk factors. Regarding the relationship between hypercholesterolemia, statin use and ICH risk, the available evidence indicates contradictory results in both primary and secondary prevention [29, 30]. Heart failure has been identified as a comorbidity but not a protective factor [31]. There is no clear interpretation of the risk of ICH in the context of heart failure or of the role of low blood pressure as a preventive factor for ICH because the data describing the optimal BP goals in patients with HF are limited and contradictory [32, 33]. However, the risk of ICH could be related to the concurrence of atrial fibrillation and a greater or lesser indication of antiplatelet and anticoagulant therapy. Neither HT nor antiplatelet therapy were recognized ICH risk factors [34, 35] that appeared explicitly in our model. Nevertheless, these are included in the HAS-BLED score, among other common bleeding risk factors. However, neither the degree of control nor the severity or duration of other cardiovascular risk factors was investigated, nor the cardiovascular risk was not measured quantitatively.

The HAS-BLED score is a useful tool for close periodic monitoring and for checking avoidable risk factors [36]. Despite its low estimated discriminative power, a HAS-BLED score ≥ 3 showed high sensitivity and a negative predictive value for the risk of ICH. This result is of special interest from a clinical point of view because it introduces the concept of “unnecessarily premature and avoidable mortality” for an episode of ICH as an indicator of the healthcare that these patients receive [18, 37]. Further research related to the sensitivity and specificity of the HAS-BLED score for the identification of ICH risk in CCPs should be performed to detect the best cut-off point for not only detection but also strategy planning. Using this approach, different HAS-BLED scores could lead to various preventive measures, which could range from optimizing HT control to deprescription of NSAIDs and antiplatelet drugs, particularly in the primary prevention of cardiovascular disease. A similar approach has been used regarding the prescription of statins to patients with a prior ICH episode [38]. Currently, there is an extensive trend towards optimization of the drug regimen of elderly patients, although a systematic and evidence-based approach has not yet been developed [39].

In this context, it is interesting to note that in the present study, 86.7% of patients with ICH and a HAS-BLED score ≥ 3 were hypertensive, and 93.3% had an active prescription of NSAIDs and/or antiplatelet drugs. The use of NSAIDs [40] and antiplatelet therapy with respect to the risk of ICH has been recognized, and evidence suggests that this effect might not be dose-dependent [41]. These drugs interact by impairing thromboxane-dependent platelet aggregation and can thus prolong the bleeding time [42]. Moreover, 53.4% of the CCP population had an active antiplatelet prescription, although only 29.2% of the patients had suffered ischaemic cardiopathy and 7.7% suffered an ischaemic stroke and/or transient ischaemic accident. The antiplatelet drugs are probably prescribed not only for secondary prevention but also for primary prevention. The benefits of antiplatelet therapy for the secondary prevention of cardiovascular disease clearly outweigh the risks of bleeding, and low-dose aspirin is consistently recommended in this setting. However, no clear consensus exists regarding whether, and if so for whom, antiplatelet therapy is appropriate for the primary prevention of cardiovascular disease, which has led to different recommendations in the organizational guidelines [43, 44]. The lack of evidence regarding the effects of these recommendations is even greater in the older population. The ACC/AHA Guidelines do not recommend aspirin as a primary prevention strategy for adults older than 70 years of age because it results in a significantly higher risk of major haemorrhage and does not result in a significantly lower risk of cardiovascular disease than placebo [45, 46]. Bleeding complications are an inherent risk in anticoagulant and thrombolytic treatments, and the benefits and risks are strongly correlated [44]. This balance leads to complex decisions, particularly in a population such as CCPs, which is defined by its complexity. Having a tool for identifying those patients with a higher risk of ICH would help improve this decision process, among others. The fact that antiplatelet drugs but not anticoagulant drugs were associated with a high risk of ICH could be due to the use of the latter in a smaller group of cases (29.2% vs 53.4%), which leads to a more difficult detection of significant differences. It is likely that the cardiovascular profile risk of those patients with prescriptions of these drugs is different, as suggested by the data in the comparative tables.

The control of comorbidities, such as cardiovascular disease, hypercholesterolemia and HT, should also be prioritized in the follow-up of CCPs. However, it should be considered that some inherent characteristics of these patients, such as institutionalization, cognitive impairment, or polypharmacy, might eventually be barriers to achieving optimal control [14].

Although patients with cardiovascular disease are expected to obtain HAS-BLED scores ≥3, the coexistence of different factors, such as advanced age, multimorbidity and polypharmacy, together with a higher incidence of ICH in CCPs would justify the individual, systematic and regular use of risk assessment tools for the detection of high-risk cases, although these might come with a high rate of false positives. Unfortunately, empirical evidence in this field is limited despite the clinical relevance of ICH and its associated adverse outcomes. This study adds to the available knowledge in an area of work that is considered very challenging due to the greater and new needs of CCPs and therefore offers an opportunity to explore clinical risk assessment modalities and prevention policies to attempt to reduce the prevalence and severity of diseases, in accordance to the suggested key points of the latest report from the European Stroke Organization, the Stroke Alliance for Europe and the World Health Organization [8, 47, 48].

Because ICH is a heterogeneous disease, this study has some limitations, such as those related to the possibility of underregistration. The information source did not allow differentiation of the type of ICH, its aetiology, or its severity. Hypertensive vasculopathy is the most common cause of spontaneous ICH, and the main factor in preventing recurrence is blood pressure control [49]. Regardless of these facts, a more accurate classification of stroke into a pathogenic subtype evades the best clinical skills when the main goal is its prevention. For this reason, ICH subtype was not considered a useful variable in the risk assessment, although a better understanding of the underlying causes of the different subtypes is needed. The lack of comparative data in relation to some factors associated with the incidence of ICH, particularly those related to antiplatelet drugs and their combination with NOACs and/or other treatments should be considered. Furthermore, our results are based on a CCP population and might not be applicable to other populations. More studies are needed to evaluate the impact on the detection of those high-risk cases when adjusted by morbidity groups [50]. Finally, the usefulness and cost-effectiveness of a risk scale in daily clinical practice related to the incidence of ICH and its consequences should be evaluated [8].

## Conclusions

- The incidence of intracerebral haemorrhage in a complex chronic patient population was 151/10,000/year (95% CI: 127–174), which is 5-to-60-fold higher than the incidence reported for the general population.
- In the population of complex chronic patients, the main risk factors associated with intracerebral haemorrhage were HAS-BLED score ≥ 3 and history of hypercholesterolemia and cardiovascular disease.
- The systematic evaluation of bleeding risk based on the HAS-BLED score or similar scores in complex chronic patients could be useful for the identification of individuals with a high risk of intracerebral haemorrhage, i.e., those who can benefit the most from optimizing the control of modifiable factors.

## Supplementary Information

**Additional file 1.**

**Additional file 2.**

## Data Availability

Currently, the datasets used and/or analysed during the current study are available from the corresponding author upon reasonable request. All data generated or analysed during this study have been deposited in the public repository: doi:10.5061/dryad.0hr311d.

## Abbreviations

BP: Blood pressure
CCP: Complex chronic patient
CI: Confidence interval
e-SAP: Electronic clinical records in primary care
CMBD: Minimum basic data set, Catalan acronym for *Conjunt Mínim Bàsic de Dades*
HT: Arterial hypertension
HF: Heart failure
ICD-10: International Classification of Diseases
ICH: Intracerebral haemorrhage
INR: International normalized ratio
IRR: Incidence rate ratio
MACA: Advanced Chronic Care Model, Catalan acronym for *Model d’Atenció a la Cronicitat Avanzada*
NOACs: New oral anticoagulants
NSAID: Nonsteroidal anti-inflammatory drugs
OR: Odds ratio
PIIC: Shared Individualized Intervention Plan, Catalan acronym for *Pla d’Intervenció Individualitzat Compartit*
SIRE: Integrated Electronic Prescription System, Catalan acronym for *Sistema Integrat* de *Recepta Electrònica*
