# Supplementary material for: Complex chronic patients as an emergent group with high risk of intracerebral haemorrhage: an observational cohort study
Source: BMC Geriatr. 2021 Feb 5;21:106. doi: 10.1186/s12877-021-02004-4 (PMC7863444; doi:10.1186/s12877-021-02004-4)
Supplement: Supplementary file 1 — Additional file 1. [file 12877_2021_2004_MOESM1_ESM.docx]

**Additional File 1**

**CCP conceptualization**

Regarding the relationship between CCPs and the health system in Catalonia (as in many European countries), CCPs have been conceptualized as a category due to their increasing number and impact on healthcare services. Approximately 4-5% of the population is estimated to be CCPs and consume 65% of the healthcare resources due to their recurrent use of health services (primary, specialized and emergency care) and multiple hospital admissions, which sometimes end in long stays. The authors have added the specific indicators used among the population identified as CCPs (see **Table)** to reflect the importance of addressing our research question in this vulnerable group aligned with the European framework. Either way, the care of patients with complex chronic conditions is a very challenging area of work due to their greater and new needs and therefore offers an opportunity to explore clinical risk assessment modalities and prevention policies.

CCPs are prescribed a broad range of drugs and exhibit low adherence to treatment, and the drugs might have side effects or interactions. In addition, patients’ loneliness, institutionalization or low social, cultural and economic levels have a great impact on the evolution of the disease. Normally, the process involves a gradual loss of independence that can have a great impact on personal, family and work levels. The prognosis of CCPs is often poor, mostly because these patients tend to have numerous symptoms that are difficult to control, and their clinical condition changes over time. Consequently, CCPs need to be identified, and their monitoring has to be adapted. The main goal is to maintain the level of health of this population as high as possible in terms of their functional status, which would result in achieving good symptom control, promoting safety aspects in pharmaceutical prescriptions, and thus reducing adverse events.

Stratification of the health risks of people with chronic diseases has been adopted in many European countries to strengthen population health management and provide better-tailored services. Two stratification models were developed in Catalonia: the first involved clinical risk groups (CRGs), and the current model includes adjusted morbidity groups (AMGs). Based on the latter, the Catalan population has been classified into different morbidity groups, and from these groups, a risk of hospitalization over the next 12 months has been set up. This work was performed thanks to the availability of the Minimum Basic Data Set at the hospital discharge register (CMBD-HA), which includes an aggregation of all minimum data sets related to primary healthcare, hospitals, nursing homes and mental health and pharmacies. The elaboration of this stratification has different functions: (a) adjustment of the simulation budgetary assignment model; (b) information included in clinical records regarding individual morbidity group classification and ‘risk scores’ related to future potential risk of hospitalization and death, which are key to the identification of CCPs and advanced chronic patients, respectively; (c) temporal and geographical distribution of morbidity; and (d) routine health indicators in individual primary healthcare records for proactive clinical decision-making, including identification of vulnerable groups, analysis of population morbidity or selection of controls for epidemiological studies.

In general terms, the Chronicity Prevention and Care Programme set up by the Health Plan for Catalonia has allowed the improvement of conditions of chronic patients and thus the achievement of better outcomes in these patients. In addition to these results, important progress in redesigning the model of care should be recognized. The combination of this care model redesign and an integrated information system has allowed a better comprehension of complex patients and their needs. New guidelines from the World Health Organization, the Stroke Alliance for Europe, and the European Stroke Organization have suggested key points for addressing diseases such as ICH among an increasing number of European people with chronic conditions.

**Table. CCP indicators included in the document "Healthcare pathway for chronicity and complexity. Terres de l'Ebre Health región”. Departament de Salut. Generalitat de Catalunya^1^. (version 6 approved June 26, 2020).**

| **1/ Prevalence indicators**   - Number of people identified as CCPs/population assigned for each primary care team. - Number of people identified as belonging to the MACA/population assigned for each primary care team. |
| --- |
| **2/ Tracking indicators**   - Number of people identified as CCPs and belonging to the MACA with PIIC (Shared Individualized Intervention Plan, Catalan acronym for *Pla d'Intervenció Individualitzat Compartit*) updated in the last year/population assigned for each primary care team. - Number of people identified as CCPs and belonging to the MACA with PIIC updated in the last year/people identified as PCC and belonging to the MACA for each primary care team. - Number of people identified as CCPs and belonging to the MACA with social assessment in the last year and an established Individual Integral Care Plan (PIAI). |
| **3/ Morbi-mortality indicators**  (Adjusted indicator and 95% CI of people identified as PCCs and belonging to the MACA for each primary care team; annual frequency)   - Mortality (per 1,000 inhabitants) - Potentially avoidable hospitalizations - Morbidity index (average weight of the adjusted morbidity groups (AMGs)) - Risk stratification (% of risk: high, moderate, low and basal) |
| **4/ Resource consumption indicators**  (Adjusted indicator and 95% CI of people identified as PCCs and belonging to the MACA for each primary care team; annual frequency)   - Number of contacts with primary care (per person). - Emergency room admissions (per 100 people) - Non-scheduled hospital admissions (per 100 people) - Long stays in a nursing home or geriatric centres (per 100 people) - People in UFISS (per 100 people): interdisciplinary socio-sanitary functional unit in hospitals that participates in counselling, evaluating patients and preparing them for discharge - People in PADES (per 100 people): home care programme and primary care support teams for both social and health problems |

^1^ document de l’ENAPISC de Procés assistencial d’atenció a la cronicitat i la complexitat a la xarxa d’atenció primària. Departament de Salut. Generalitat de Catalunya. Disponible en:

http://salutweb.gencat.cat/web/.content/_ambits-actuacio/Linies-dactuacio/Estrategies-de-salut/enapisc/enapisc-cronicitat-complexa.pdf
