# Supplementary material for: Complex chronic patients as an emergent group with high risk of intracerebral haemorrhage: an observational cohort study
Source: BMC Geriatr. 2021 Feb 5;21:106. doi: 10.1186/s12877-021-02004-4 (PMC7863444; doi:10.1186/s12877-021-02004-4)
Supplement: Supplementary file 2 — Additional file 2. [file 12877_2021_2004_MOESM2_ESM.docx]

**Additional File 2**

| **Primary care teams in the health area of Terres de l’Ebre, Catalonia, Spain (2020). Institut Català de la Salut.** |
| --- |
| 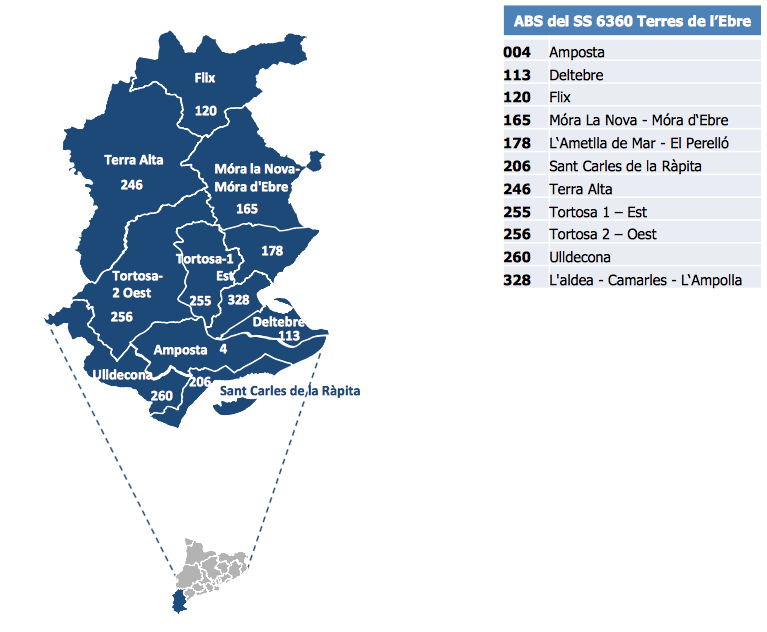 |
| Sectors sanitaris dividits per ABS [Internet]. [cited 2020 Jan 27]. Available from: <https://catsalut.gencat.cat/web/.content/minisite/catsalut/coneix_catsalut/transparencia/docs/mapa-ss-abs.pdf> |
